# Supplementary material for: Postharvest Application of Acibenzolar-S-Methyl Activates Salicylic Acid Pathway Genes in Kiwifruit Vines
Source: Plants (Basel). 2023 Feb 13;12(4):833. doi: 10.3390/plants12040833 (PMC9962033; doi:10.3390/plants12040833)
Supplement: Supplementary file 1 [file plants-12-00833-s001.zip › plants-2207603-supplementary.pdf]

Table S1. DNA sequence of the capture and reporter probes used to analyse kiwifruit gene expression by PlexSet® Nanostring.

Gene name	Gene ID	Function	Capture probe	Reporter probe
Eukaryotic small ribosomal subunit 40S	40S	Reference	CTACAAGCTCCTT GGTGGCCTCGCTG TTCGCAGGGCCTG CTATGGCGTTT	TGAGAT TTGTTATGGAGAGC GGGGCAAAGGGAT GTGAGGTGATTGTT AGT
Ubiquitin-conjugating enzyme	UBC	Reference	ATCTGAACGATTA CTCACATCCACAG AATCGACCATTTC AGGAACAAAAA	AATCCCC TCCAACAATTCACT GGCCTGATCGACGA TCTAATTCTTCTCCG
Glyceraldehyde 3-phosphate dehydrogenase	GAPDH	Reference	ACTTTGTTGGTGAC AGCAGATCGAGCA TCTTTGATGCCAAG GCTGGGATT	GCTTTG AACGACTTGTTCGT GAAACTGGTGTCTT GGTATGACAACGAG TG
Protein phosphatase 2	PP2A	Reference	TCCAGAATGGGCA ATGCAGCACATAA TTCCACAGGTATTG GACATGATTA	GCAACC CACATTATCTGTAC CGTATGACCATACT ACACTCGATCTCTC TT
Pathogenesis-related protein family 1	*PR1*	Target gene	GTTTGTGGGCACT ACACTCAAATTGT GTGGAGAAACTCG GTCCGGCTCGG	GTGCGC TAGGGTTCGGTGCA ATAGTGGGTCTTGG TTCGTTACTTGCAA CT
APETALA2 ethylene responsive factor 2	*AP2_ERF2*	Target gene	TTGGCCTATGACA GGGCGGCTTTTAG TATGCGTGGGGCG AAGGCTCTCCT	CAATTT TCCAGCTGAAGTAG GGGCAGAATCGTCC AAGCAAAGATTTAC CC
Glucan endo-1,3-β-glucosidase	*PR2*	Target gene	TGCTTGTGATTTCC CTCATAAAGAGGG CACTAGCAAAAAA TAGAGTATGT	ACCGAGA GATTGCTCCTATGA AGACAGACAAAAT ATCTAATAAAGGAA TA
Thaumatin-like protein TG4	*PR5*	Target gene	AATATCATAAACA ACTGCCCTTTCACC GTTTGGGCCGCTG CCGTTCCAGG	TGGTGG CAAACGCCTTGACC GTGGCCAGAATTGG ATCATCAATCCTGG TG
NIM-interacting protein 2	*NIMIN2*	Target gene	AGCGGAGCGATGA CGTGGAGGCCGAC GCCAAGAAGGCG AGGGTAGGGGAA	GATA ACGGAAAAGTGAC GGAGCCGGAGGAC GATGAGGTGGAGG AGTTCTT
Downy mildew resistance 6	*DMR6*	Target gene	ACGCCCTCACAAT TTTGCTTCAGGACC	CTCAAGGACGGCA AGTGGATGGCCGTC

			TCCAAGTCTCAGG CCTACAAGTC	AAACCCCATCCCAA TGCCTTTGT
WRKY transcription factor 70	*WRKY70*	Target gene	TGGAGGAAATATG GACAAAAGGAGAT CCTCAATGCCAAA TTTCCAAGGTG	CTA CTTTAGGTGCACAC ACAAGCCTGATCAA GGTTGCCTAGCAAC AAAGC
Benzyl alcohol dehydrogenase	*BAD*	Target gene	GCCGATATAGAGC TGATTCCGATGGA CTATGTGAACACC GCGATGGAGCG	GCTTGT GAAGGCTGACGTTA GTCCCTTGAGGCAT TTTGGGCTTGACAA GC
